# Natural biomass-derived carbon dots as a potent solubilizer with high biocompatibility and enhanced antioxidant activity

**DOI:** 10.3389/fmolb.2023.1284599

**Published:** 2023-11-02

**Authors:** Tong Wu, Menghan Li, Tingjie Li, Yafang Zhao, Jinye Yuan, Yusheng Zhao, Xingrong Tian, Ruolan Kong, Yan Zhao, Hui Kong, Yue Zhang, Huihua Qu

**Affiliations:** ^1^ School of Chinese Materia Medica, Beijing University of Chinese Medicine, Beijing, China; ^2^ School of Traditional Chinese Medicine, Beijing University of Chinese Medicine, Beijing, China; ^3^ School of Life Science, Beijing University of Chinese Medicine, Beijing, China; ^4^ Centre of Scientific Experiment, Beijing University of Chinese Medicine, Beijing, China

**Keywords:** carbon dots, solubilization, biomass, antioxidant, nanocomplex

## Abstract

Numerous natural compounds exhibit low bioavailability due to suboptimal water solubility. The solubilization methods of the modern pharmaceutical industry in contemporary pharmaceutical research are restricted by low efficiency, sophisticated technological requirements, and latent adverse effects. There is a pressing need to elucidate and implement a novel solubilizer to ameliorate these challenges. This study identified natural biomass-derived carbon dots as a promising candidate. We report on natural fluorescent carbon dots derived from *Aurantia Fructus Immatures* (AFI-CDs), which have exhibited a remarkable solubilization effect, augmenting naringin (NA) solubility by a factor of 216.72. Subsequent analyses suggest that the solubilization mechanism is potentially contingent upon the oration of a nanostructured complex (NA-AFI-CDs) between AFI-CDs and NA, mediated by intermolecular non-covalent bonds. Concomitantly, the synthesized NA-AFI-CDs demonstrated high biocompatibility, exceptional stability, and dispersion. In addition, NA-AFI-CDs manifested superior free radical scavenging capacity. This research contributes foundational insights into the solubilization mechanism of naringin-utilizing AFI-CDs and proffers a novel strategy that circumvents the challenges associated with the low aqueous solubility of water-insoluble drugs in the field of modern pharmaceutical science.

## 1 Introduction

In pharmaceutical development, over 40% of newly synthesized candidate therapeutics fall under BCS II or BCS IV, categories notorious for their poor water solubility (Charalabidis et al., 2019), particularly compounds originating from natural plants. These plant-derived derivatives (e.g., paclitaxel, resveratrol, and curcumin) have demonstrated a plethora of functional diversity and therapeutic potential, finding applications in the food industry, biopharmaceuticals, and adjacent sectors (Li and Vederas, 2009; Kundu et al., 2021; Cruz-Hernández et al., 2022). Nevertheless, these compounds all exhibit poor bioavailability due to their low aqueous solubility. Water remains the predominant solvent, and the aqueous solubility of these compounds constitutes a crucial determinant influencing their efficacy, which in turn constrains their subsequent advancement. Recently, a myriad of solubilization strategies have been employed to enhance aqueous solubility, such as the modification of hydrophobic groups, augmentation of the glycosidic proportion, encapsulation via amphiphilic molecules, and incorporation of surfactants (Kometani et al., 1996; Jambhekar and Breen, 2016; Liang X. et al., 2023; Wang X. et al., 2023). However, these methodologies have yielded suboptimal solubilization outcomes and the unintended release of noxious chemical agents, potentially culminating in toxicity.

Naringin (NA), a ubiquitous therapeutic employed in the food and pharmaceutical sectors, is comprised of 4′,5,7-hydroxyflavone (saccharide ligand) and rhamnose-β-1,2-glucose and is one of the most abundant bioflavonoids in citrus fruits (Ghanbari-Movahed et al., 2021; Jiang et al., 2023). Regarded as a critical therapeutic agent with a broad spectrum of physiological effects, NA not only exhibits potent bioactivities in anti-inflammatory (Bharti et al., 2014), anti-osteoporosis (Gan et al., 2023b), and anticancer modalities (Farhan, 2022), but also amplifies the absorption of other therapeutics (Choi and Shin, 2005). As a common compound in BCS II, NA possesses poor water solubility, which is measured at 81.1 μM (4.71 × 10^−2^ g/L) at 25°C in the pH range of 1–7.6. This escalates formulation costs and impacts drug absorption in the intestine (Rao et al., 2017; Xiang et al., 2021). Numerous solubilization techniques exist to enhance its solubility, including structural modification, liposome formation, and polymeric micelle encapsulation (Ravetti et al., 2023). Nevertheless, these approaches often entail intricate experimental conditions and hazardous reagents with prohibitive costs and stringent synthetic constraints; furthermore, commonly synthesized complexes exhibit low encapsulation efficiency and compromised stability (Jat et al., 2022; Secerli et al., 2023). Thus, this body of evidence has catalyzed our pursuit to conceive a novel strategy to address these limitations.

Carbon dots (CDs), recognized as emerging zero-dimensional carbonaceous materials, are spherical nanodots comprising an sp^2^ or sp^3^ carbon core and complex surface oxygen-containing groups (Nair et al., 2020; Wareing et al., 2021; Gan et al., 2023a). CDs manifest an ultrasmall size, high biocompatibility, chemical robustness, and notable bioactivities, rendering them promising candidate biomaterials for biotherapy (Wang L. et al., 2023; Kalluri et al., 2023; Singh et al., 2023). Previous studies have corroborated that CDs possess considerable potential for development and utilization in the field of solubilization. CDs can not only adsorb compounds via complex porous surfaces (Cutrim et al., 2021), but also bind small molecules through surface functional groups and charge accumulation via non-covalent interactions (e.g., electrostatic interactions, hydrogen bonding, and coordinate bonds) (Arcudi and Đorđević, 2023). Concomitantly, these complexes engendered by CDs and drugs exhibit low biotoxicity, high drug-loading efficiency, and sustained release profiles (Kaurav et al., 2023; Veerapandian et al., 2023). However, the synthesis of many CDs typically involves chemical precursor materials and noxious reagents, which could impede their further biomedical applications. Propitiously, a considerable array of CDs utilizing low-cost and readily available green materials as precursors have been synthesized via environmentally benign processes, while retaining many of the functional groups of the precursor and sufficient bioactivities (Nair et al., 2020; Luo et al., 2021). It is noteworthy that herbal medicine, classified as a potent green precursor, is increasingly garnering attention due to its high yield, abundant active components, and verified pharmacological activities among natural biomass precursors (Luo et al., 2022; Qiang et al., 2023). Simultaneously, some herbal medicine-derived CDs have demonstrated excellent solubilization effects (Luo et al., 2019; Zhang et al., 2021b), and these mechanisms remain largely unexplored.

**FIGURE 1:**
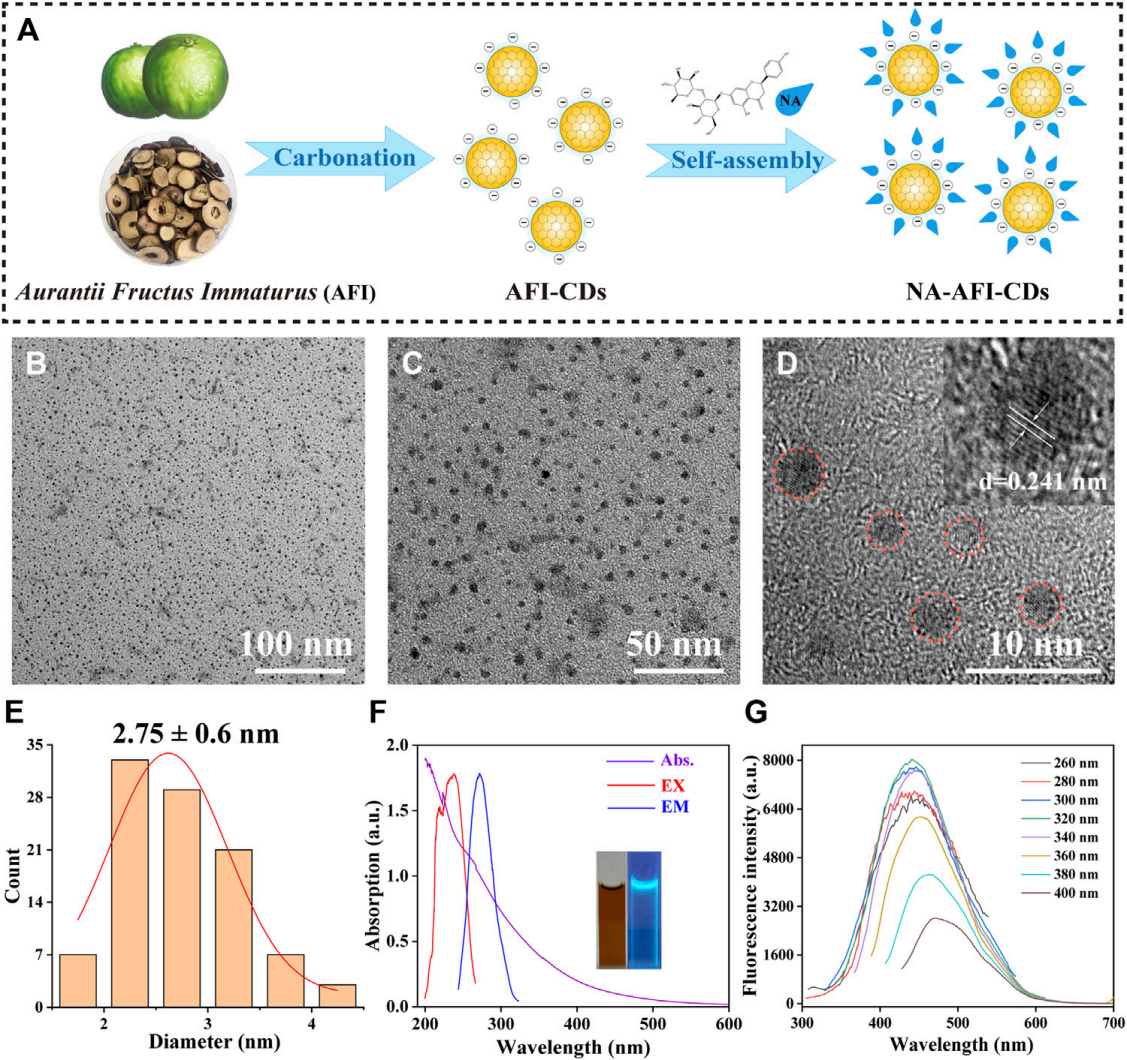
Synthesis and characterization of AFI-CDs. **(A)** Schematic diagram of AFI-CDs and NA-AFI-CDs. **(B, C)** TEM images of AFI-CDs at 100 and 50 nm. **(D)** High-resolution TEM image of AFI-CDs (inset, lattice space of AFI-CDs). **(E)** Diameter distribution of AFI-CDs measured by ImageJ. **(F)** UV-vis and FL spectra (inset, AFI-CDs solution in light and UV light). **(G)** Fluorescence spectra of AFI-CDs with different excitation wavelengths. a.u., arbitrary units.

Thus, motivated by processing technology from Traditional Chinese Medicine (TCM), we synthesized a green natural CD (named AFI-CDs) derived from *Aurantia Fructus Immatures* (an immature fruit from citrus) via a simple one-step pyrolysis method (Figure 1A). We then ascertained its superior solubilization effect on NA compared with other herb-derived CDs (Supplementary Table S1). Subsequently, we devised and optimized a preparation method using AFI-CDs to enhance the solubility of NA and postulated that the solubilization mechanism likely hinges upon the formation of a nanostructured complex (NA-AFI-CDs) between AFI-CDs and NA. Predicated on a series of characterization and property tests, NA-AFI-CDs were revealed to engage in specific structural assembly mediated by intermolecular non-covalent forces. Additionally, AFI-CDs and NA-AFI-CDs both exhibited marked biocompatibility and antioxidant capacities. This study harbors the potential to facilitate the development of an innovative solubilizer for NA and contribute to the foundational theory of solubilization via herb-derived CDs.

## 2 Results and discussion

### 2.1 Synthesis and characterization of AFI-CDs

As illustrated in (Supplementary Figure S1), AFI-CDs synthesized via a one-step pyrolysis method underwent a green synthesis process and incorporated no additional pharmaceutical ingredients, thereby ameliorating the complications associated with convoluted operations and unstable efficacy in synthesis. Following dialysis, we procured transparent brown stock solutions, and their freeze-dried powders exhibited remarkable water solubility. The outcome of the high-performance liquid chromatography (HPLC) analysis concerning raw AFI and AFI-CDs demonstrated that no small molecules (such as naringin or hesperidin) remained in the AFI-CDs solution, thereby eliminating interference from uncarbonized natural compounds (Supplementary Figure S2).

Utilizing transmission electron microscopy (TEM), AFI-CDs manifested uniformly spherical nanoscale dots with excellent dispersibility (Figures 1B, C). The majority exhibited a narrow particle size distribution of 1–5 nm with an average diameter of approximately 2.75 ± 0.6 nm, as ascertained through quantitative analysis and statistical methods employing ImageJ software (Figure 1E). The micrograph in high-resolution TEM verified a graphite-like crystalline structure with a lattice spacing of 0.241 nm (Figure 1D), congruent with the common lattice spacing of carbon skeletons in CDs derived from other natural products (Zhai et al., 2023). In accordance with the recombination of radiation on the surface functional groups, numerous CDs possess distinctive optical characteristics and luminescence (Liu et al., 2022; Tao et al., 2023). Spectral analyses of AFI-CDs revealed weak π-π* electronic transitions in the sp^2^ domains at approximately 280 nm in the UV-vis spectrum (Lu et al., 2021), and their maximal emission and excitation were observed at 320 nm and 440 nm, respectively, in fluorescence spectra (Figure 1F). Simultaneously, the aqueous solution of AFI-CDs (depicted in the inset image in Figure 1F) emitted a light blue-green fluorescence under UV light at 365 nm. Additionally, AFI-CDs displayed excitation-dependent emission behaviors as the excitation wavelength varied from 260 to 400 nm at 20 nm intervals (Figure 1G). Collectively, these indicators substantiate that AFI-CDs are ultrasmall (<5 nm) CDs with fluorescence.

### 2.2 Solubilization effects of AFI-CDs

**FIGURE 2:**
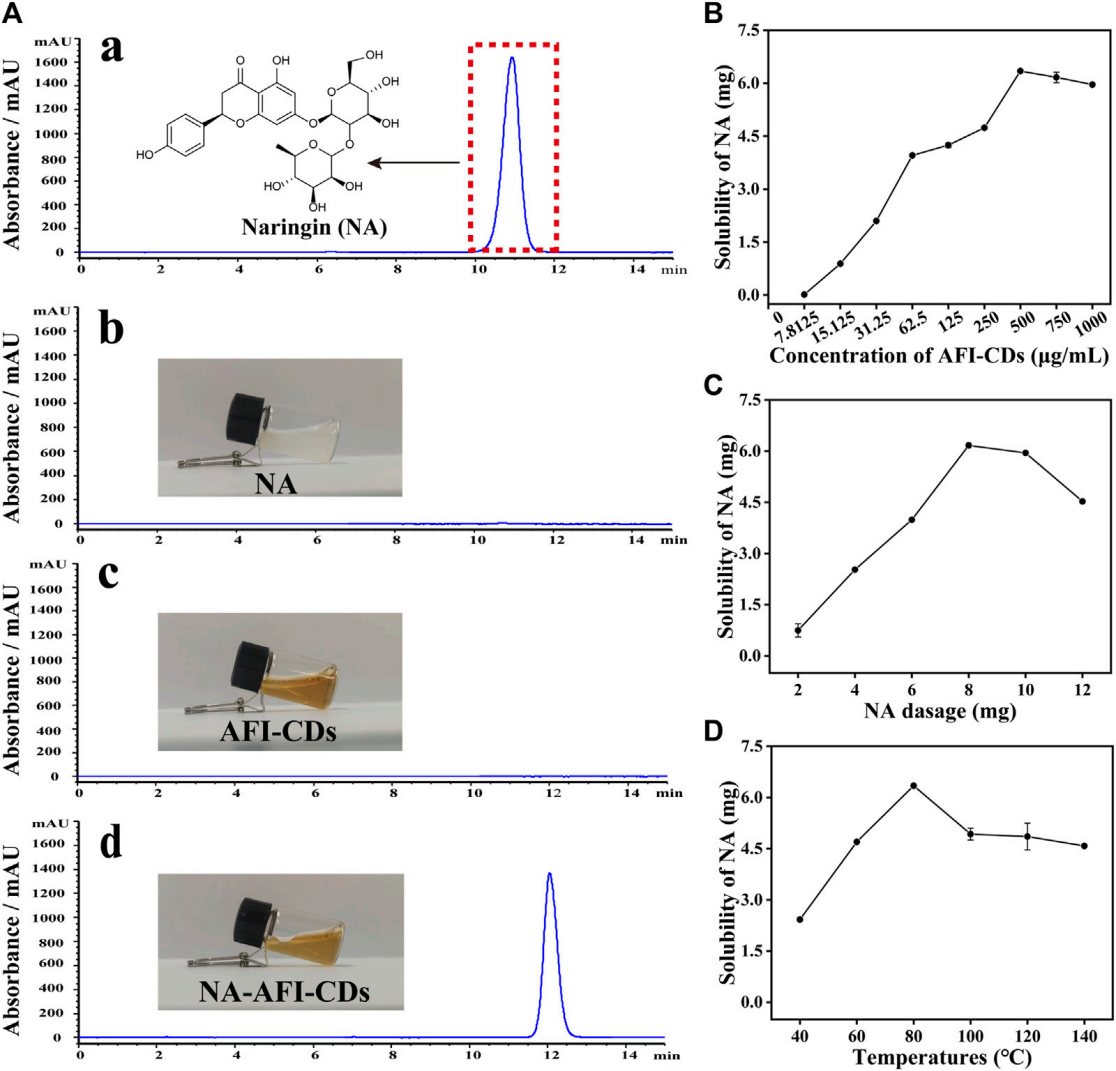
Solubilization tests of AFI-CDs. **(A)** HPLC chromatograms of **(a)** NA in methanol; **(b)** NA in water; **(c)** AFI-CDs; and **(d)** NA-AFI-CDs. **(B–D)** The effect of the concentration of AFI-CDs, NA dosage, and temperature. Each set of experiments was repeated three times.

The HPLC chromatograms of NA and AFI-CDs indicated that the selectivity of the established HPLC method exhibited commendable performance for subsequent investigation (Figure 2A). The residual standard deviations (RSDs) of precision, repeatability, and stability amounted to 0.64%, 0.60%, and 0.94%, respectively. The linear regression equation of the standard curve was formulated as: Y = 7020.2X+233.25, *R* ^2^ = 0.9999, where Y and X denote the HPLC map peak area and concentration of NA (mg/mL). The linear range of NA spanned from 9.9275 mg/mL to 0.019 mg/mL, and all analytical parameters fell within the methodological requirements.

Previously ascertained process parameters exerted a substantial influence on the solubilization of small molecular compounds (You et al., 2021; Tian et al., 2022). Our study concentrated on augmenting the value of NA through interaction with AFI-CDs. As the concentration of AFI-CDs escalated from 7.8125 to 500 μg/mL, the solubility of NA experienced a pronounced increase from 0.01631 mg/mL to 6.3449 mg/mL, subsequently declining at 750 μg/mL (Figure 2B), a phenomenon consistent with previous studies on the solubilization of CDs (Chen et al., 2023). The dosage of NA was an integral determinant influencing the stability of the NA-AFI-CDs. As the dosage of NA increased from 2 to 12 mg, the solubility of NA initially proliferated up to 8 mg, then diminished (Figure 2C). The temperature of the aqueous solution constituted an important factor in NA-AFI-CDs; as illustrated in Figure 2D, the solubilization of NA correlated positively with temperature and amounted to 2.42, 4.70, and 6.34 mg at temperatures of 40, 60, and 80°C, respectively. No discernible differences in solubility were observed from 100°C to 140°C, and all registered lower concentrations of NA than the procedure conducted at 80°C. Concomitantly, we investigated variations in solubility attributable to heating duration; minimal fluctuations were evident with heating from 4 to 10 h. Thus, the optimal heating duration for the solubilization of AFI-CDs transpired to be 6 h in the aqueous solution (Supplementary Figure S3). Additionally, we examined the impact of varying oscillation durations on the solubilization of NA. As oscillation time extended, the solubilization of NA escalated from 3.31 to 6.34 mg and manifested a notable decrease at 160 min, suggesting that protracted oscillations may compromise the stabilization of NA-AFI-CDs (Supplementary Figure S4). Simultaneously, we analyzed and compiled DLE and SE of NA-AFI-CDs under various conditions and observed a congruent trend with the solubility of NA (Supplementary Tables S2−S6). In accordance with the empirical findings, the optimal preparation conditions for NA-AFI-CDs were ascertained: 8 mg of NA (13.73 μmol) was amalgamated with 500 μg/mL of AFI-CDs in a vial, a mix that was subjected to agitation for 80 min at room temperature; subsequently, the procured solution underwent heating at 80°C for 4 h in the dark. The optimal method was deemed applicable for ensuing research endeavors.

### 2.3 Solubilization mechanism of AFI-CDs

#### 2.3.1 Morphology of NA-AFI-CDs

**FIGURE 3:**
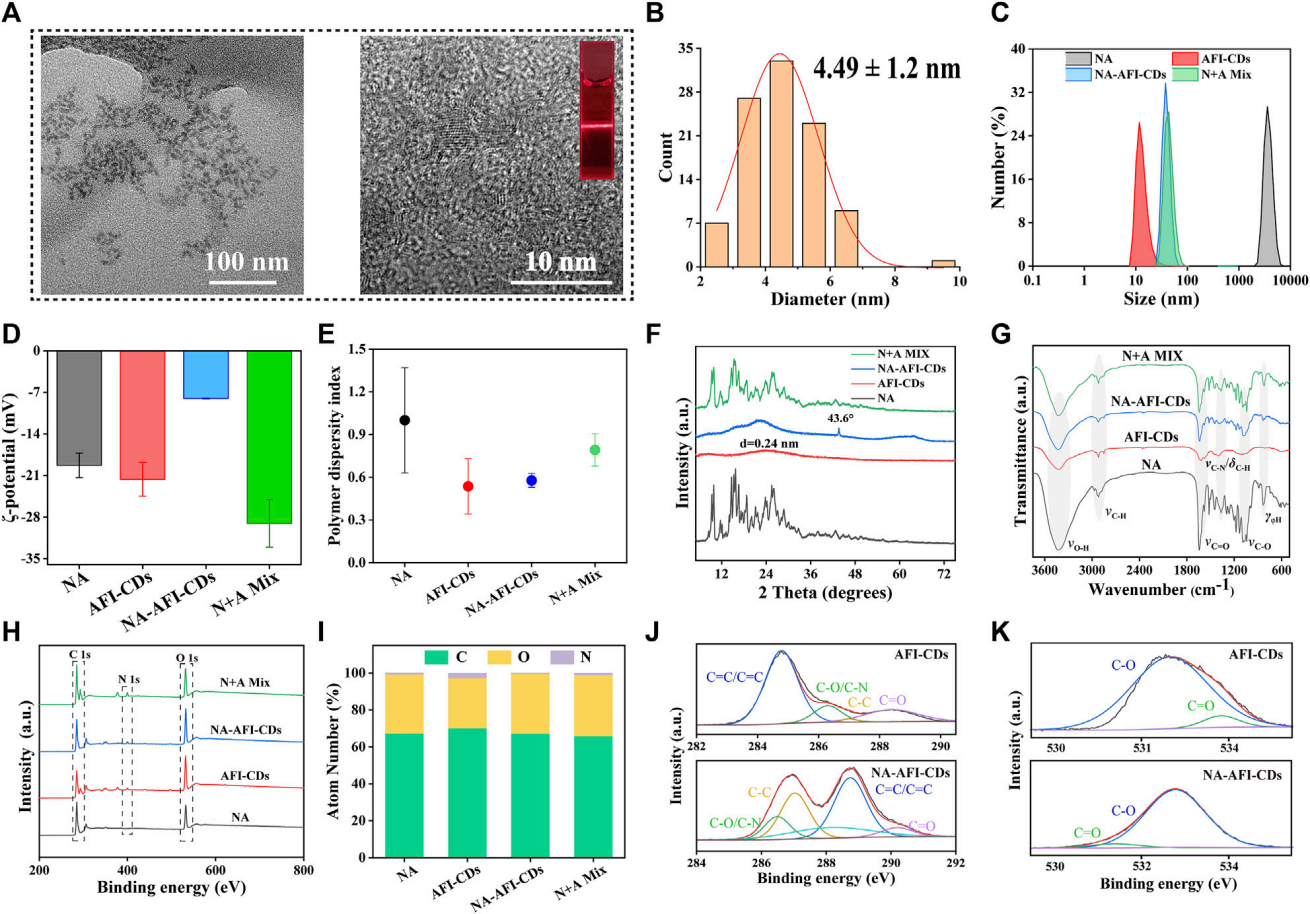
Morphology and characterization of NA-AFI-CDs. **(A)** TEM and HRTEM of NA-AFI-CDs (inset, Tyndall phenomenon of AFI-CDs). **(B)** Diameter distribution of NA-AFI-CDs. **(C)** DLS assay. **(D)** ζ-potentials evaluation. **(E)** PDI assay. **(F)** XRD spectra. **(G)** FT-IR spectra. **(H)** XPS spectra. **(I)** Elemental composition. **(J)** C 1s high-resolution XPS spectra. **(K)** O 1s high-resolution XPS spectra. a.u., arbitrary units.

As depicted in the inset image in Figure 2A, the aqueous solution of AFI-CDs manifested as a lucid and transparent solution with a pH of 6.7, whereas NA constituted a milky suspension in water, evidencing weak solubility in water. Conversely, the solution of NA-AFI-CDs exhibited a pellucid brown hue, akin to the AFI-CDs solution, implying that insoluble NA may transmute into an amorphous state during interaction with AFI-CDs. Figure 3A, acquired through TEM and HRTEM, reveals that NA-AFI-CDs comprised agglomerated spherical nanodots devoid of discernible lattice space. Figure 3B indicates that NA-AFI-CDs possessed a homogeneous shape with an augmented mean particle size of 4.49 ± 1.2 nm, ranging from 2 to 10 nm, when compared with the AFI-CDs solution. Simultaneously, in comparison with NA suspension, the Tyndall phenomenon of NA-AFI-CDs manifested as a narrow bright singular light path, corroborating the colloidal nature of the solution (Figure 3A and Supplementary Figure S5). Moreover, dynamic light scattering (DLS) ascertained that the NA suspension exhibited poor distribution (PDI = 1) and an approximate hydrodynamic size of 3,000 nm, attributable to its low water solubility. Contrastingly, both AFI-CDs and NA-AFI-CDs displayed uniform distribution in water, with hydrodynamic diameters of 11.7 and 38.8 nm, respectively; their uniform PDI indicated optimal distribution in the water dispersion system (Figures 3C, E). The ζ-potentials of NA, AFI-CDs, NA-AFI-CDs, and the physical mixture were measured as −19.3 ± 2.07, −21.6 ± 2.81, −8.0 ± 0.05, and −29.1 ± 4.01 mV, respectively (Figure 3D). The negative charge was correlated with abundant oxygen-containing groups, and the enhanced charge of NA-AFI-CDs was ascribed to electrostatic adsorption (Ghanbari et al., 2021).

#### 2.3.2 Optical nature of NA-AFI-CDs

To elucidate the altered optical characteristics of the nanostructure, the synthesized AFI-CDs and NA-AFI-CDs samples were characterized via UV-vis absorption spectrophotometer and fluorescence (FL) spectroscopy, respectively. UV-vis absorption spectrophotometer further validated the interaction of NA-AFI-CDs **(** Supplementary Figure S6A **)**. Peaks at 223.46 and 282.9 nm were identifiable as the characteristic peaks of NA, and the UV spectrum of NA-AFI-CDs paralleled that of NA. The absorbance of NA-AFI-CDs underwent a blue shift from 224.6 to 223.4 nm, suggesting that small molecules and CDs may amalgamate to form a complex. Intriguingly, NA-AFI-CDs exhibited diminished blue-green fluorescence relative to AFI-CDs and the physical mixture of AFI-CDs and NA (Supplementary Figure S6B). Concurrently, FL spectra discerned that NA-AFI-CDs emanated weaker emission fluorescence with a redshift from 438 to 449 nm (Supplementary Figure S6C). These phenomena imply that NA-AFI-CDs are not a mere mixture of AFI-CDs and NA but rather a complex composite of the two.

#### 2.3.3 Characterization of NA-AFI-CDs

The elucidation of the formulation mechanism of NA-AFI-CDs was predicated upon a series of spectroscopic characterizations. As depicted in Figure 3F, the X-ray diffraction patterns (XRD) of the NA exhibited multiple sharp diffraction peaks with a well-defined crystal structure. The integral amorphous state of AFI-CDs was preserved due to its elevated aqueous solubility. Concurrently, the reflection planes at approximately 26.603° were attributed to highly disordered carbon structures corresponding to the (002) plane of the graphitic framework (Xu et al., 2022). Furthermore, NA-AFI-CDs manifested a high-energy amorphous state, obliterating the characteristic peaks and introducing sharp peaks related to the correlation with the amorphization of the NA. The physical mixture exhibited a hybrid characterization between NA and AFI-CDs, corroborating that the novel nanostructure was successfully synthesized rather than being a mere physical mixture.

Subsequently, Fourier Transform Infrared Spectrometry (FT-IR) validated the formation of NA-AFI-CDs (Figure 3G). NA-AFI-CDs possessed analogous chemical signatures to those of NA and AFI-CDs, such as γ-C-OH (3,420 cm^-1^) of AFI-CDs and γ-C=O (1,618 cm^-1^) and γ-C-O (1,100 cm^-1^) of NA, as well as NA-AFI-CDs exhibiting a predominant spectrum closely resembling AFI-CDs. In contradistinction, the physical mixture of NA and AFI-CDs comprised solely the sample superposition of monomer materials. This evidence substantiates that NA and AFI-CDs were successfully amalgamated. Moreover, the absorption peaks such as γ-C-N (from 1,404 to 1,385 cm^-1^) and γ-C-O (from 1,088 to 1,081 cm^-1^) exhibited slight shifts to lower wavenumbers with attenuated peak intensities, compared with those of AFI-CDs and NA, respectively. This phenomenon is attributable to electrostatic interaction and intermolecular hydrogen bonding between NA and AFI-CDs, resulting in the weakening of the chemical bond force constant and delocalization of the electron cloud (Pinilla-Peñalver et al., 2022).

Subsequent X-ray Photoelectron Spectroscopy (XPS) was undertaken to augment the structural assessment, and the resultant spectra of each element were documented (Figure 3H). The elemental composition in Figure 3I and Supplementary Table S7 revealed that NA-AFI-CDs contain a higher proportion of O (32.47%) and a diminished proportion of N (0.2%) compared with NA (O, 32.01%; N, 0.59%) and AFI-CDs (O, 27.27%; N, 0.2%), signifying that oxygen elements play an integral role in the assembly of NA-AFI-CDs. The XPS survey energy spectrum furnished additional corroborative evidence of bonding (Figures 3J, K, Supplementary Figures S7, S8). The C 1s spectrum of AFI-CDs consisted of three surface components manifesting at 284.80, 286.30, 281.02, and 288.39 eV, corresponding to C=C/C≡C, C-O/C-N, C=C, and C=O, respectively (Tong et al., 2020). The distinctive binding energy spectrum of C=C/C≡C and C-C indicated the amorphous character of AFI-CDs with a carbon core comprising both sp^2^ and sp^3^ C orbitals (Li et al., 2023). The core level spectrum of O 1s in the top exhibited two components with binding energies at 532.73 and 533.87 eV, attributed to C=O and C-OH bonds (Liang P. et al., 2023). Pertaining to NA-AFI-CDs, the XPS survey spectrum of NA-AFI-CDs displayed similar characteristic peaks for C, O, and N elements as observed in the unmodified AFI-CDs and NA, and altered binding energies of distinct chemical bonds indicated interactions between the two materials. For instance, the XPS spectrum of the C=C bond of C 1s exhibited an elevated value in NA-AFI-CDs compared with NA and AFI-CDs, which may be correlated with amorphous NA interacting with AFI-CDs through a series of processes. All the characterization results revealed that the solubilization mechanism is primarily attributed to the formation of cluster-like amorphous nanocomplexes between NA and AFI-CDs; concurrently, non-covalent forces (e.g., electrostatic adsorption and intermolecular hydrogen bonding) play a pivotal role in the interlacing of the two materials.

#### 2.3.4 The interaction between NA and AFI-CDs

**FIGURE 4:**
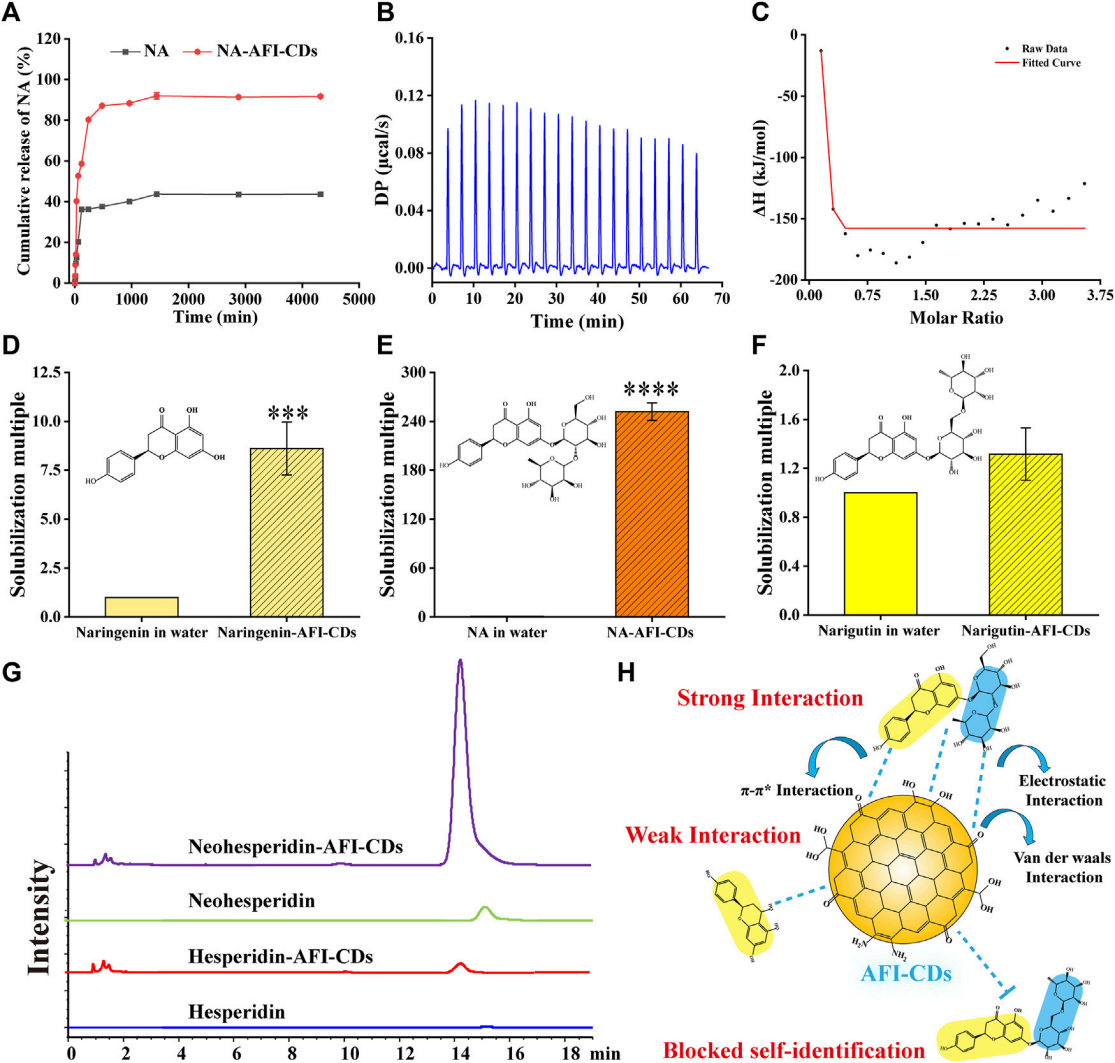
The interaction evaluation and solubilization mechanism of NA-AFI-CDs. **(A)** *In vitro* release profile of NA and NA-AFI-CDs. **(B)** The calorimetric titration. **(C)** Binding isotherm. **(D–F)** The solubilization effect assays to naringenin, naringin, and narirutin. **(G)** HPLC chromatograms of hesperidin and neohesperidin mixed with water or AFI-CDs. **(H)** Schematic diagram of the solubilization mechanism.

Although the aforementioned evidence affords sufficient insight to substantiate structural alterations and crystal transition, it lacks direct corroboration to further elucidate intricate aspects of the interaction between NA and AFI-CDs. The *in vitro* release profile constitutes a classical methodological approach for ascertaining the binding state and delivery capacity of the complex. In Figure 4A, a saturated NA solution, employed as a control group, attained its cumulative drug release in 1,440 min at 43.69%. By contrast, the cumulative drug release from NA-AFI-CDs not only reached 91.96% within the same identical release timeframe but also exhibited a lower dissolution rate than the control group. These outcomes indubitably substantiate that the solubilization capability of AFI-CDs possesses substantial potential for drug release and affirms that the interaction between NA and AFI-CDs is primarily governed by non-covalent forces rather than covalent bonds. Subsequently, isothermal titration calorimetry (ITC) was used to explore the presence of non-covalent forces between NA and AFI-CDs. An AFI-CDs solution was titrated with an NA suspension at room temperature (25°C), manifesting a positive energy change between the two constituents, thereby suggesting an exothermic reaction propelled by an enthalpy change (Figure 4B). Concomitantly, the negative ∆H and -T∆S signified the spontaneity and stability of this reaction (Figure 4C) (Zhao et al., 2020). These findings corroborate that non-covalent forces facilitate aggregation and self-assembly between NA and AFI-CDs.

Moreover, the solubilization efficacy of AFI-CDs on naringenin, NA, and narirutin was assessed. The data demonstrated that AFI-CDs augmented the solubilization efficacy of naringenin eight-fold compared with its solubility in water, thereby corroborating that the interaction of the glycosidic fraction of NA with AFI-CDs has a pronounced impact on its solubilization capability (Figures 4D, E). Pertaining to narirutin, an isomer of NA with the identical glycoside, AFI-CDs exhibited no significant solubilization effect (Figure 4F), implying that AFI-CDs may achieve enhanced solubilization through the recognition and binding to corresponding glycosidic structures. To validate this hypothesis, the solubilization efficacy of AFI-CDs on hesperidin and neohesperidin (isomers with identical glycosides) was assessed; the findings indicated a stronger solubilization effect for neohesperidin than for hesperidin (Figure 4G). The neohesperidin possesses a comparable disaccharide structure and binding site with NA, indicating that AFI-CDs principally recognize these disaccharides and self-assemble into complexes to enhance solubility. This molecular recognition phenomenon may be facilitated by non-covalent forces generated between these AFI-CDs and NA (Figure 4H). Extant literature confirms that CDs significantly inhibit the conformational change of isomers through self-recognition (Liang et al., 2022; Jv et al., 2023), and our data elucidate that such recognition also amplifies molecular interactions and facilitates assembly into complexes. Owing to limited techniques, AFI-CDs lack a well-defined chemical structure for identification. Our analyses have thus far scrutinized the principal nanostructure of NA-AFI-CDs and partially assessed interactions between molecules, necessitating further verification through robust research methodologies in the future.

### 2.4 Physicochemical properties of NA-AFI-CDs

**FIGURE 5:**
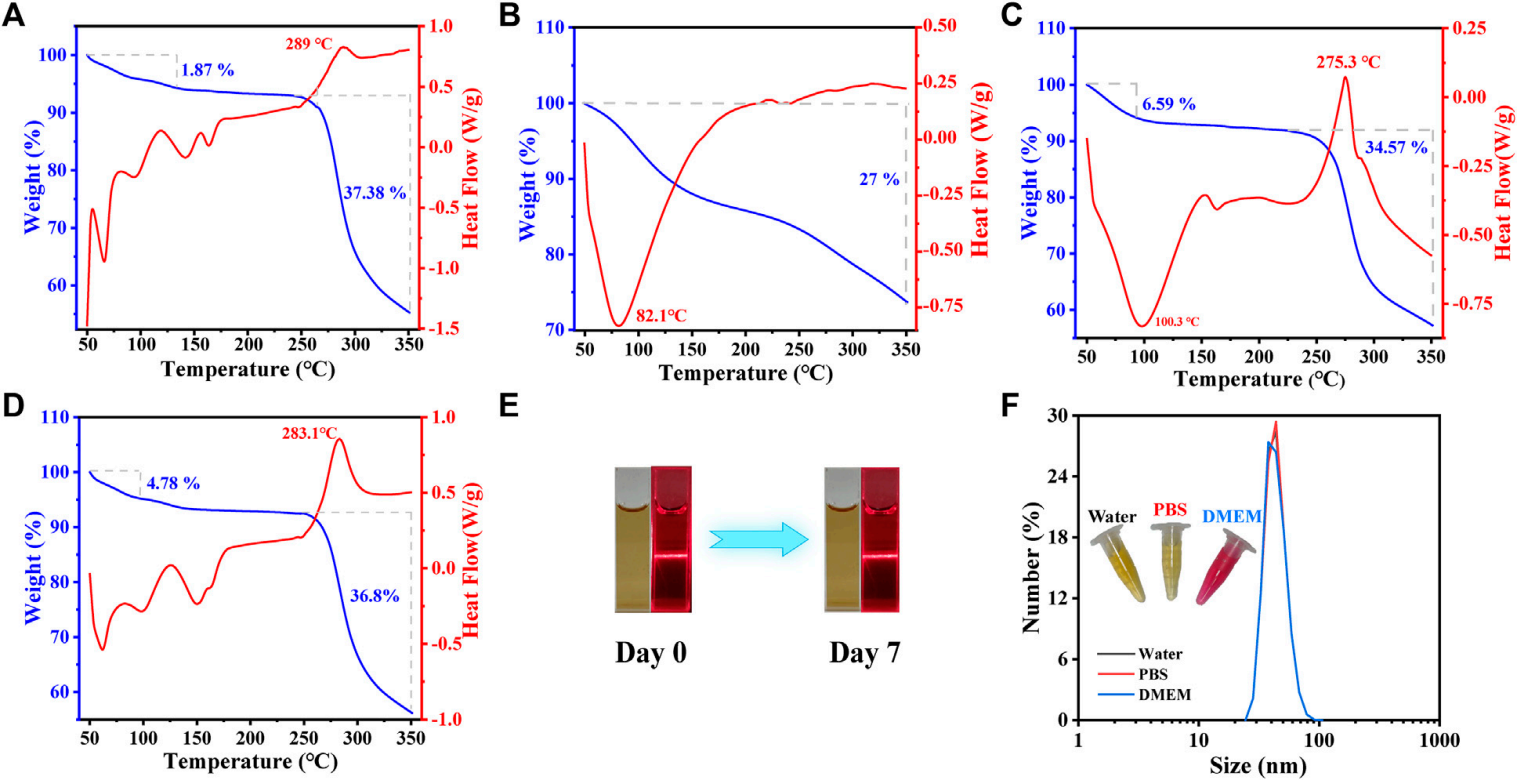
Physicochemical properties of AFI-CDs. The thermal assay by TG (blue line) and DSC (red line) on NA **(A)**, AFI-CDs **(B)**, NA-AFI-CDs **(C)** and physical mixture **(D)**. The stability of NA-AFI-CDs were investigated at room temperature **(E)** and with different solvents (water, PBS, and DMEM) **(F)**.

To assess the thermal stability of NA-AFI-CDs, TG (thermogravimetry) and DSC (differential scanning calorimetry) analyses were conducted under an N_2_ atmosphere. As illustrated in Figure 5A, the initial phase of pure NA in TG revealed a weight loss of 5.65% from 50°C to 135°C, attributed to the loss of adsorbed water and crystalline water. In the second phase, a substantial weight loss (37.38%) from 248°C to 350°C was observed, attributable to the thermal degradation of NA. In the DSC curve, NA manifested a distinct endothermic enthalpy commencing at 248°C, correlated with the structural decomposition induced by the melting of NA crystals. AFI-CDs exhibited only a singular weight loss zone (27%) from 50°C to approximately 350 °C, correlated with water evaporation (Figure 5B). In the DSC curve for AFI-CDs, a pronounced broad exothermic peak at 82.1°C suggested the decomposition of crystalline water in the carbon dots during the heating process. The data for NA-AFI-CDs indicated relative stability between 50°C and 225°C, accompanied by a gradual weight loss of 34.57% from 225°C to 350°C (Figure 5C). For NA-AFI-CDs, the DSC thermogram revealed the characteristics of both NA and AFI-CDs, with the characteristic peak of NA shifting to 230°C, indicating the formation of a novel structure between NA and AFI-CDs. TG and DSC thermograms of the physical mixture yielded similar mass loss and residual mass relative to NA, suggesting that the simple physical mixture exerted no significant influence on thermogravimetric alterations (Figure 5D). The TG curves disclosed that the four samples possessed 55.29%, 73.66%, 57.25%, and 56.17% thermal decomposition residues, corresponding to NA, AFI-CDs, NA-AFI-CDs, and the physical mixture, respectively. The expanded pyrolysis temperature range and higher proportion of residual substances implied that NA-AFI-CDs boasted superior thermal stability.

Furthermore, the solvent stability of NA-AFI-CDs was evaluated. The aqueous solution of NA-AFI-CDs presented a clear brown fluid with a narrow light path characteristic of the Tyndall phenomenon and retained its clarity without sedimentation even after 7 days at room temperature (Figure 5E). Concurrently, the hydrodynamic size demonstrated exceptional stability over a 7-day period in water, phosphate-buffered saline (PBS), and cell culture medium (Dulbecco’s Modified Eagle’s Medium or DMEM) (Figure 5F). Collectively, these data substantiate that NA-AFI-CDs exhibit exceptional stability.

### 2.5 Biocompatibility of AFI-CDs and NA-AFI-CDs

**FIGURE 6:**
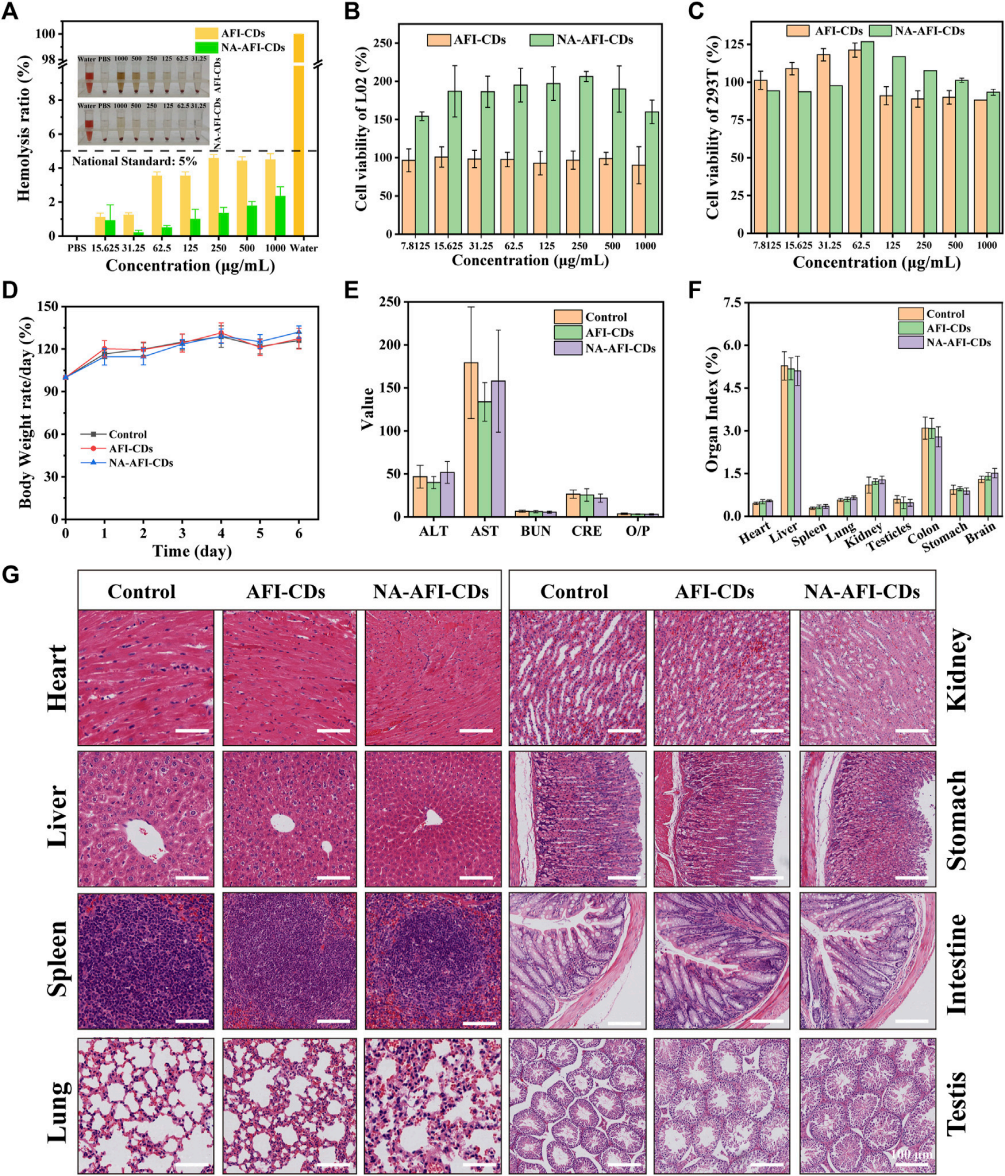
Biosafety evaluation of AFI-CDs and NA-AFI-CDs. **(A)** The hemocompatibility test of NA-AFI-CDs at different concentrations. **(B, C)** Cell viability of L02 and 293T cells with AFI-CDs and NA-AFI-CDs, respectively (*n* = 6). **(D)** Body weight change in the control, AFI-CDs, and NA-AFI-CDs groups (*n* = 6). **(E)** Biochemical data. **(F)** Organ index. **(G)** Histological evaluation of eight major organs of mice in different groups on day 7. Scale bars: 100 μm. Data are mean ± SD. *p < 0.05.

Numerous solubilizers exhibit varying degrees of toxicity and potential side effects. Despite the eco-friendly synthesis process of AFI-CDs and NA-AFI-CDs, it is imperative to consider the potential biotoxicity arising from possible interactions with biological systems (Domingues et al., 2000; Zhang et al., 2022). A hemolysis assay, cell cytotoxicity test, and biosafety evaluations were conducted *in vitro* and *in vivo*, respectively. The hemolysis assay results showed that even at concentrations of AFI-CDs and NA-AFI-CDs as high as 1,000 μg/mL, there was negligible hemolytic activity in rat red blood cells (Figure 6A). The solutions of all groups were clear, and the chromatic attributes of NA-AFI-CDs were all comparable with that of the PBS group. The solutions of all groups were clear, and the chromatic attributes of the NA-AFI-CDs solution were all comparable with that of the PBS group. Concomitantly, the hemolysis rates of AFI-CDs were significantly lower than internationally recognized standards (5%), indicating their well biocompatibility (Jia et al., 2022). The cytotoxicity of AFI-CDs and NA-AFI-CDs was assessed in L02 and 293T cells, respectively. When the concentration of AFI-CDs increased to 1,000 μg/mL, cell viability for both L02 and 293T cells remained above 80%, corroborating the low cytotoxicity of CDs as previously documented (Zhang et al., 2021a). For NA-AFI-CDs, the survival rates of L02 cells exceeded 100% from concentrations ranging from 1,000 μg/mL to 7.8125 μg/mL (Figure 6B), suggesting that NA-AFI-CDs may facilitate L02 growth due to the enrichment of hepatocytes by nanoparticles. Simultaneously, all concentrations of NA-AFI-CDs manifested negligible toxicity on 293T cells (Figure 6C). As previously reported (Li et al., 2022), a myriad of CDs derived from carbonized plants exhibited negligible cytotoxicity across most concentrations, and NA-AFI-CDs, a composite of CDs and NA, maintained low cytotoxicity. These aforementioned results imply that AFI-CDs and NA-AFI-CDs possess very low biotoxicity and could be amenable to further *in vivo* evaluations.

Additionally, further assessments concerning potential toxicity toward biochemical indices and multiple organs *in vivo* were undertaken. After administration of AFI-CDs and NA-AFI-CDs for 7 days, there were no appreciable weight changes compared with the control group (Figure 6D). Concurrently, nanoparticles might accumulate and potentially occlude the liver or kidneys through metabolic and excretory processes, yielding alterations in biochemical indices (Vilas-Boas and Vinken, 2021). As depicted in Figure 6E, biochemical indices related to liver and kidney function (ALT, AST, BUN, CRE, and O/P) exhibited no significant changes upon administration with AFI-CDs or NA-AFI-CDs, mirroring *in vitro* cellular experiments. Given that CDs might induce inflammation in major organs and gastrointestinal injuries (Zhang et al., 2023), toxicity assessments for five major organs (heart, liver, spleen, lung, and kidney) and digestive organs (stomach and intestine) were also conducted. Nanoscale carbon materials have been suspected to cause reproductive toxicity or brain injury (Hansen and Lennquist, 2020; Kang et al., 2023); thus, our study also observed pathological changes in the testes and brain. As illustrated in Figures 6F, G and Supplementary Figure S9, no appreciable organ index changes between the control and administration groups were detected, nor were any significant morphological or pathological abnormalities. In summary, according to these results, AFI-CDs and NA-AFI-CDs demonstrated high levels of biocompatibility and biosafety *in vitro* and *in vivo*.

### 2.6 Antioxidant ability of AFI-CDs and NA-AFI-CDs

**FIGURE 7:**
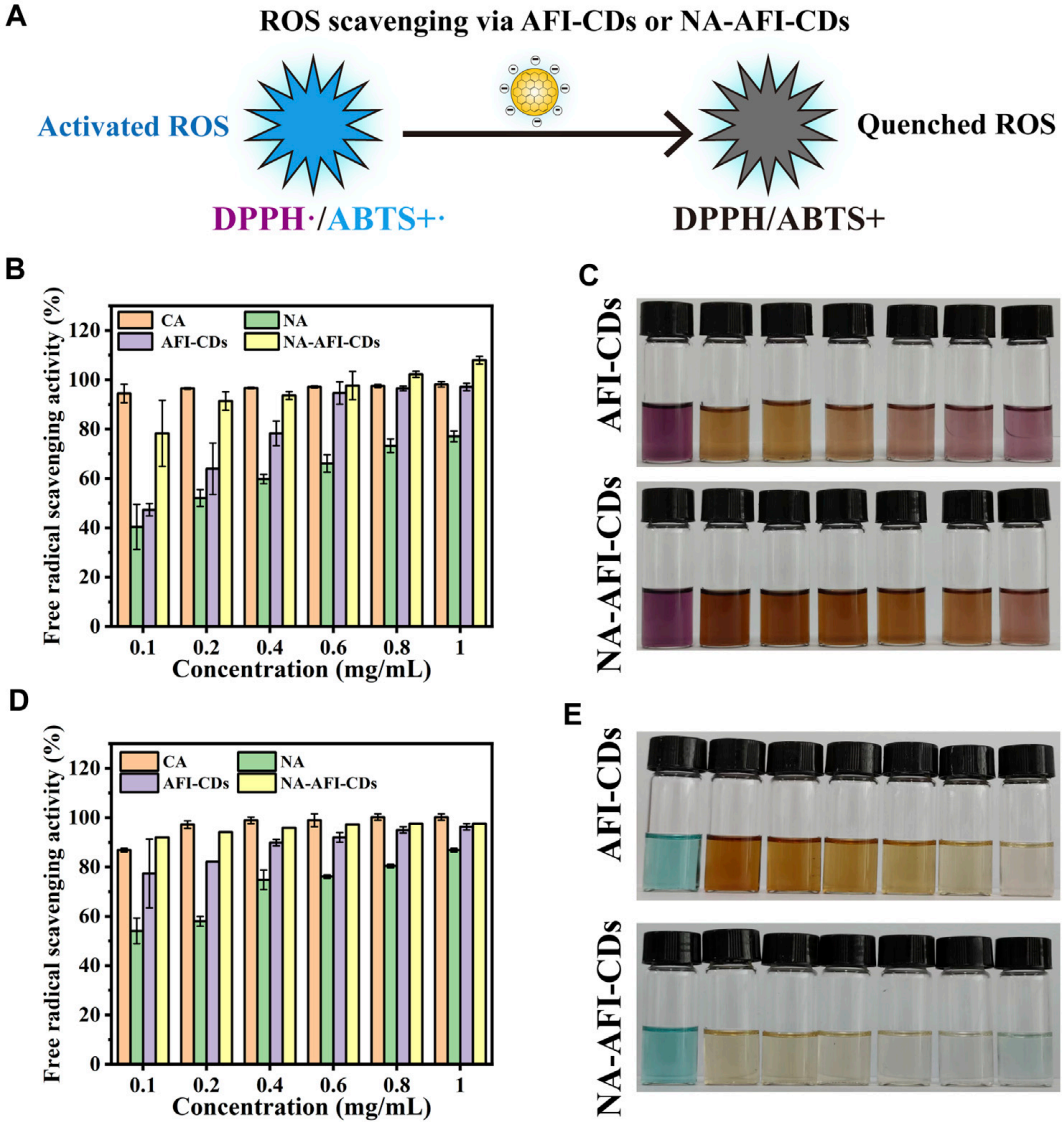
Antioxidant assays of AFI-CDs and NA-AFI-CDs. **(A)** Schematic illustration of the ROS scavenging process. **(B)** DPPH radical scavenging ability of CA, NA, AFI-CDs, and NA-AFI-CDs. **(C)** A photograph of the reaction systems featured in **(B)**. **(D)** ABTS+• radical scavenging ability of CA, NA, AFI-CDs, and NA-AFI-CDs. **(E)** A photograph of the reaction systems featured in **(D)**.

Numerous studies have ascertained the formation between CDs and polyphenols (such as lutein and naringenin) can significantly enhance antioxidant efficacy (Pérez-Gálvez et al., 2020) (Figure 7A). In the present study, 1,1-diphenyl-2-picrylhydrazyl (DPPH, a stable nitrogen-centered free radical) and 2,2′-azinobis-(3-ethylbenzthiazoline-6-sulphonate) (ABTS) were used to investigate the oxidative resistance of these nanomaterials (Liu et al., 2021; Han et al., 2023). Citric acid (CA) was employed as a control group to evaluate antioxidative capabilities. As illustrated in Figure 7B, the results for AFI-CDs and NA-AFI-CDs manifested dose-dependency and enhanced DPPH• scavenging activities compared with pure NA. Ranging from 0.1 to 0.6 mg/mL, the DPPH• scavenging rate of AFI-CDs incrementally augmented, while the free radical scavenging rate remained analogous to that of CA beyond that concentration. The NA-AFI-CDs exhibited robust antioxidant potency, comparable with CA at low concentrations (0.4 mg/mL) and surpassing it at elevated concentrations (*p* < 0.01). As paramagnetic entities with solitary electrons, DPPH• free radicals accept an electron from a free radical scavenger to synthesize a stable DPPH-H compound (Zhu et al., 2004), inducing a colorimetric transition from dark purple to the respective solution color. After incubation, AFI-CDs and NA-AFI-CDs both manifested efficacious clearance effects, as depicted in Figure 7C.

Moreover, ABTS can be oxidized by K_2_S_2_0_8_ to yield the cationic radical ABTS+, a solution with a blue-green hue; the presence of antioxidants reverts them back to their original ABTS form. Upon interaction with AFI-CDs or NA-AFI-CDs, the chromaticity of the ABTS+• solution progressively attenuated to either colorless or the inherent hue of the nanomaterial solution; these changes were concomitantly observed with increasing concentrations of AFI-CDs or NA-AFI-CDs (Figure 7E). Relative to radical scavenging proficiency, AFI-CDs virtually obliterated ABTS+• at 1 mg/mL, and NA-AFI-CDs achieved a commensurate scavenging impact at 0.6 mg/mL (Figure 7D). However, CA possessed a superior clearance rate of approximately 100% at 0.8 mg/mL. The putative antioxidant mechanism underlying the augmentation of antioxidant capabilities in NA-AFI-CDs consisted of the complexation between NA and AFI-CDs, which facilitated electron or hydrogen atom transfer and contributed additional electron donors to neutralize free radicals (Chae et al., 2021). With the increment in NA-AFI-CDs, the antioxidant efficacy markedly amplified, which could be attributed to the combinatorial synergistic interplay between NA and AFI-CDs.

## 3 Materials and methods

### 3.1 Materials

Naringenin, naringin, narirutin, hesperidin, neohesperidin, and citric acid were procured from Chengdu Herbpurify Biotechnology Co., Ltd (Chengdu, China), and the purity of these compounds exceeded 98%. The sample of AFI (Batch No: 220708002) was acquired from Beijing Qiancao Traditional Chinese Medicine Co., Ltd (Beijing, China), and AFIC (*Aurantii fructus immaturus* carbonisata) was synthesized in our laboratory. HPLC-grade methanol was procured from Honeywell (New York, USA). The remaining analytical-grade chemical reagents were sourced from Sinopharm Chemical Reagents Beijing (Beijing, China). Fetal bovine serum (FBS), Dulbecco’s modified Eagle’s medium (DMEM), and cell counting kit (CCK-8) were acquired from Beijing BioDee Biotechnology Co., Ltd (Beijing, China). All experiments were conducted using deionized water (DW).

### 3.2 Animals

Male Kunming (KM) mice (SPF-group, 8-week-old, 25.0 ± 2.0 g) were procured from SIBEIFU Biotechnology Co., Ltd. (Beijing, China). All animals were accommodated in a temperature-controlled environment (20°C–25°C) with a relative humidity of 50%–60% and a 12 h light/dark cycle. We adhered to all guidelines of the laboratory animal center. All animal procedures in this study complied with the Guide for the Care and Use of Laboratory Animals and were endorsed by the Animal Ethical Committee of Beijing University of Chinese Medicine.

### 3.3 Synthesis of AFI-CDs and NA-AFI-CDs

AFI-CDs were synthesized via a modified one-step pyrolysis method (Wang et al., 2019). Concisely, 200 g of dry AFI was placed in crucibles, and encased with aluminum foil. Subsequently, the crucibles were sustained at a temperature of 350°C for 1 h for additional carbonization in a muffle furnace (TL0612, Beijing Zhong Ke Aobo Technology Co., Ltd.; Beijing, China). Post-pyrolysis, the prepared AFIC was ground into powder and subjected to a water bath at 100°C twice for 1 h each. The resultant mixture dispersions were filtered using a 0.22 μm cellulose acetate membrane. Thereafter, the filtered brown dispersions were subsequently concentrated and dialyzed using DW for 72 h. Ultimately, AFI-CDs were freeze-dried in a freeze dryer (TGL-16G, Beijing Restorative Centrifuge Manufacturing Plant, Beijing, China) for 3 days. The prepared AFI-CDs were redispersed in DW to achieve solutions of varying concentrations. NA was incorporated into the AFI-CDs solution. The resultant mixture was homogenized on the shaker and continuously heated at a constant temperature. To mitigate interference from residual NA, the synthesized solution, after cooling to ambient temperature, was centrifuged (4,000 rpm) for 10 min. The supernatant was isolated and freeze-dried to yield dry pure powder (named NA-AFI-CDs). Equivalent quantities of AFI-CDs and NA were co-mingled to fabricate the physical mixture, serving as the control. All samples were preserved at 4°C for subsequent utilization.

### 3.4 Characterization of AFI-CDs and NA-AFI-CDs

Morphology, size distribution, and thickness were assessed via Transmission Electron Microscopy (TEM, Tecnai G220) and high-resolution TEM (HRTEM, JEN-1230) at 220 kV. The UV-vis absorption spectra were acquired on a UV-vis spectrophotometer (CECIL, United Kingdom), and the FL spectra were scrutinized via a fluorescence spectrophotometer (F-4500, Japan). The DLS and ζ-potential were ascertained by Malvern Zetasizer Nano ZS (Zetasizer Nano ZS 90, United Kingdom) at 25°C. FT-IR spectra were recorded on a Fourier transform infrared spectrophotometer (Thermo Fisher, United States) to ascertain surface structure. X-ray diffraction patterns (XRD, D8-Advanced, Germany) with Cu Kα radiation (*λ* = 1.5418 Å) were used to assess crystalline alterations. X-ray photoelectron spectroscopy (XPS, Thermo Fisher Scientific, United States) and elemental composition were executed with a monochromatic Al Kα X-ray source. The thermal characteristics of the samples were assessed via thermogravimetry (TG) and differential scanning calorimetry (DSC). TG curves were concurrently acquired using an SDT-Q600 thermal analyzer from room temperature to 350°C. A METTLER TOLEDO thermal analyzer was employed for deriving DSC curves with respect to time. These analyses were conducted under a nitrogen atmosphere (20 mL/min) at a heating rate of 10°C min^−1^.

### 3.5 Solubilization experiment

#### 3.5.1 Naringin determination

The anhydrous NA standard was accurately weighed and aliquoted into methanol to fabricate a stock solution with a concentration of 9.9275 mg/mL. Subsequently, a spectrum of dilutions was prepared to yield solutions ranging from 0.019 to 9.9275 mg/mL. The quantification of NA was performed via HPLC (Agilent LC-1260, Waldbronn, Germany) employing a C-18 column (250 mm × 4.6 mm × 0.5 μm, ZORBAX SB-C18, United States). The mobile phase comprised pure water (A) and methanol (B), and isocratic elution was instituted with a constituent ratio of 65% phase A from 0 to 20 min. The injection volume, flow rate, column temperature, and detection wavelength were 10 μL, 1 mL/min, 30°C, and 284 nm, respectively. Additional methodological validation was performed in accordance with repeatability, precision, and stability. Moreover, all samples were filtrated through a 0.22 μm cellulose membrane for purification and analyzed in triplicate. The solubility of NA, drug loading efficiency (DLE), and solubilization effect (SE) were employed to gauge solubilization efficacy, and DLE and SE were calculated using the ensuing equations:

$$DLE\;\left(\%\right)=\frac{Loaded\;NA\;dosage\;\left(mg\right)}{Total\;NA\;dosage\;\left(mg\right)}\times 100$$

$$SE\;\left(fold\right)=\frac{Peak\;area\;of\;simples}{Peak\;area\;of\;NA\;in\;water}$$

#### 3.5.2 Solubilization test

To examine the influence of disparate process conditions, a suite of experiments was conducted employing a single-variable approach, encompassing variables such as the concentration of AFI-CDs, dosage of NA, oscillation time, heating duration, and heating temperature. The synthesis was executed in darkness to ensure that the samples were devoid of photonic interference. Each batch of sample was consistently positioned throughout the process.

#### 3.5.3 *In Vitro* release profile of NA

Release profiles utilizing dialysis methods were executed to assess solubilization behavior *in vitro*. Concisely, NA-AFI-CDs (5 mL) and saturated NA suspension (5 mL, comprising 8 mg NA) were independently loaded into dialysis bags (MW = 1,000 Da), after immersion in freshly prepared PBS solution (pH = 6.8) containing 0.1% Tween 80, and magnetically stirred at 180 rpm and 37°C ± 2°C for 72 h. Thereafter, an equivalent volume of sample was replaced at designated sampling intervals (5, 10, 20, 30, 60, 120, 240, 480, 960, 1,440, 2,880, and 4,320 min), and all samples were triply analyzed after filtration through a 0.22 μm filter membrane.

#### 3.5.4 The interaction assay by ITC

In accordance with preceding investigations concerning nanocomplexes, isothermal titration calorimetry (ITC) analysis curves were generated to substantiate non-covalent interactions between compounds (Pi et al., 2023). An AFI-CDs suspension (8 mg NA dispersed in 3 mL DW) was introduced into the sample cell; concomitantly, an NA suspension was allocated to the injection syringe and the reference cell contained deionized water. To circumvent bubble formation during titration, all samples were meticulously degassed for 30 min. All ITC experiments were conducted at room temperature. The NA suspension was titrated into the sample cell in 20 individual injections, each comprising 2.5 μL. Each injection yielded a singular peak in the isotherm. The angular velocity of the injection syringe was 250 rpm.

### 3.6 Biosafety evaluation

#### 3.6.1 Cytotoxicity assays

The CCK-8 assay was performed on L02 cells (normal hepatocyte) and 293T (human renal epithelial cell) to measure the cytotoxicity of AFI-CDs and NA-AFI-CDs. Briefly, cells were cultured on a 96-well plate at 37°C and 5% CO_2_, which spread to 1×10^4^ per well for 24 h. Then, 100-μL aliquots of medium containing different concentrations of nanoparticles (1,000, 500, 250, 125, 62.5, 31.25, 15.63, and 7.81 μg/mL) were added to each well for 24 h, and 10% CCK-8 solution was added to each well for an additional 2 h after cleaning three times with PBS. The absorbance of each well was recorded using a microplate reader (BioTek, Vermont, United States). The cell viability was calculated according to the following formula:

$$Cell\;viability\;\left(\%\right)=\frac{A_{e}-A_{c}}{A_{b}-A_{c}}\times 100,$$

where Ae, Ab, and Ac represent the absorbance of the experimental, control, and blank (no cells) groups at 450 nm, respectively.

#### 3.6.2 Hemocompatibility *in vitro*

Red blood cells, isolated from fresh rat blood, were used to fabricate a 10% erythrocyte suspension in PBS. A microplate reader was employed to quantify the absorbance of materials, measured at 570 nm and averaged over three calculations. The positive control comprised DW and the negative control consisted of PBS. The following formula was employed to compute the hemolysis rate:

$$Hemolysis\;rate\;\left(\%\right)=\frac{D_{a}-D_{b}}{D_{c}-D_{b}}\times 100,$$

where Da is the hemolysis absorbance of the experimental group, Db is the hemolysis absorbance of the negative control group, and Dc is the hemolysis absorbance of the positive control group.

#### 3.6.3 Biosafety evaluation *in vivo*

After administering AFI-CDs and NA-AFI-CDs intragastrically at a dosage of 30 g/kg for 7 days, peripheral blood samples were harvested into centrifuge tubes via ocular extraction and were employed to ascertain hematological parameters using an automated biochemical analyzer (AU-480, Beckman Kurt Co., Ltd., Brea, CA, United States). Simultaneously, the principal organs were weighed and subjected to histological examination with H&E staining in accordance with standard procedures.

### 3.7 Antioxidant study

The free radical scavenging abilities of DPPH• and ABTS+• were the important indices to evaluate the antioxidant activity of nanoparticles. Simultaneously, ABTS+• solution was synthesized by amalgamating 0.8 mL ABTS (4 mg/mL) and 1 mL potassium persulfate (K_2_S_2_O_8_, 1 mg/mL) and allowing them to incubate overnight in the dark. Likewise, a DPPH• solution (0.04 g/L) was formulated in an anhydrous ethanol medium under dark conditions. Typically, dried powders of CA, NA, AFI-CDs, and NA-AFI-CDs were diluted to various concentrations (0.1, 0.2, 0.4, 0.6, 0.8, and 1 mg/mL). Subsequently, 1 mL of each sample was admixed with 1 mL of DPPH• or ABTS+• anhydrous ethanol (constituting the experimental cohort). The resultant solution was sequestered in darkness for 30 min. A mixture of DPPH• or ABTS+• ethanol solution with 1 mL of DW served as the blank control, and the comparative control group was constituted by samples to which an equal volume of anhydrous ethanol had been added. The inhibition percentage and free radical scavenging efficacy were calculated employing relevant equations.

$$Radical\;scavenging\;activity\;\left(\%\right)=1-\frac{A_{0}-A_{1}}{A_{2}}\times 100,$$

where A_0_ is the absorbance of the experimental group, A_1_ is the absorption of the control group, and A_2_ is the absorbance of the blank group.

### 3.8 Statistical analysis

Results are presented as mean ± SD. Student’s t-test or one-way analysis of variance (ANOVA) were used to analyze statistical significance between two or multiple groups, respectively. Differences were considered to be statistically significant when *p* < 0.05.

## 4 Conclusion

In summation, AFI-CDs, unique green multifunctional CDs synthesized from AFI, were successfully fabricated and demonstrated excellent solubilization efficacy for naringin (216.72-fold) by assembling into NA-AFI-CD complexes without the utilization of any auxiliary agents for the first time. The procured NA-AFI-CDs were elucidated by TEM images, DLS analysis, ζ-potential measurements, UV-vis spectra, PLFL spectra, FT-IR spectra, XRD spectra, and XPS spectra, and these results revealed that the composition of NA-AFI-CDs predominantly hinges on intermolecular non-covalent bonds. Simultaneously, these forces drive AFI-CDs to more readily recognize specific glycoside structures. The procured NA-AFI-CDs exhibited exceptional stability and dispersibility, as well as manifested high biocompatibility *in vitro* and *in vivo*. The antioxidant assays substantiated that NA-AFI-CDs outperformed in free radical scavenging capacity when compared with pure NA and AFI-CDs, signifying a synergistic effect engendered by interactions between NA and AFI-CDs. This utilization of AFI-CDs may pave the way for a novel solubilization strategy pertaining to naringin in food or pharmaceutical domains. Future studies shall concentrate on the synthesis of additional herbal-derived CDs to function as solubilizers and further elucidate details in bio-delivery systems.

## Data Availability

The original contributions presented in the study are included in the article/Supplementary Material, further inquiries can be directed to the corresponding authors.
