# Supplementary material for: Natural biomass-derived carbon dots as a potent solubilizer with high biocompatibility and enhanced antioxidant activity
Source: Front Mol Biosci. 2023 Nov 2;10:1284599. doi: 10.3389/fmolb.2023.1284599 (PMC10652762; doi:10.3389/fmolb.2023.1284599)
Supplement: Supplementary file 1 [file Table1.docx]

Supplementary Material

Natural biomass-derived carbon dots as potent solubilizer with high biocompatibility and enhanced antioxidant activity

**Tong Wu^1^, Menghan Li^2^, Tingjie Li^1^, Yafang Zhao^2^, Jinye Yuan^2^, Yusheng Zhao^2^, Xingrong Tian^2^, Ruolan Kong^2^, Yan Zhao^2^, Hui Kong^2^*, Yue Zhang^3^*, Huihua Qu^2,4^***

^1^ School of Chinese Materia Medica, Beijing University of Chinese Medicine, Beijing 100029, People’s Republic of Chinese

^2^ School of Traditional Chinese Medicine, Beijing University of Chinese Medicine, Beijing 100029, People’s Republic of Chinese

^3^ School of Life Science, Beijing University of Chinese Medicine, Beijing 100029, People’s Republic of Chinese

^4^ Center of Scientific Experiment, Beijing University of Chinese Medicine, Beijing 100029, People’s Republic of Chinese

*** Correspondence:**

[doris7629@126.com](mailto:doris7629@126.com) (Hui, Kong); 201801024@bucm.edu.cn (Yue, Zhang); quhuihuadr@163.com (Huihua, Qu).

# Supplementary Figures


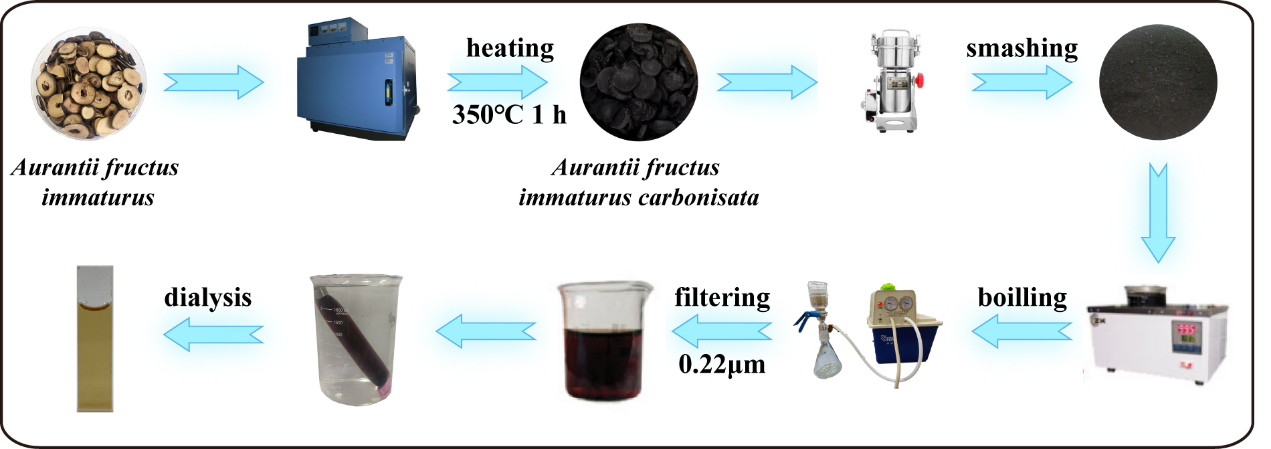


**Figure S1.** The synthesis process of AFI-CDs.


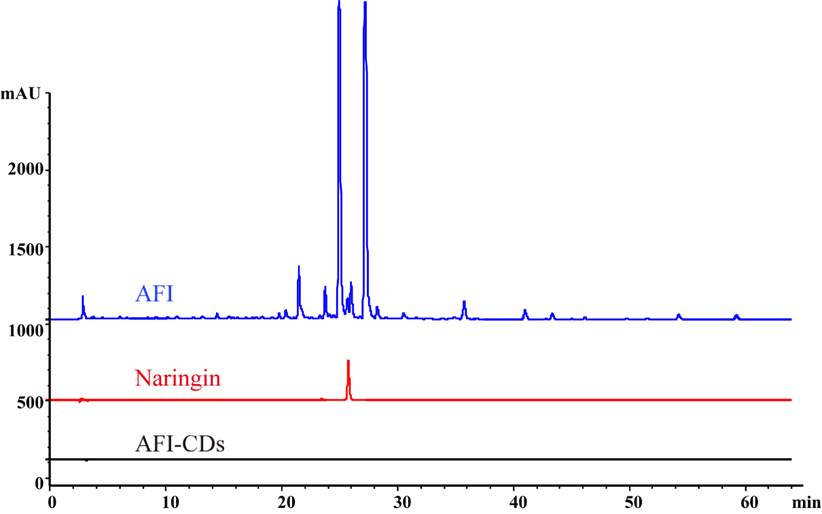


**Figure S2.** High-performance liquid chromatography (HPLC) of AFI, naringin and AFI-CDs.


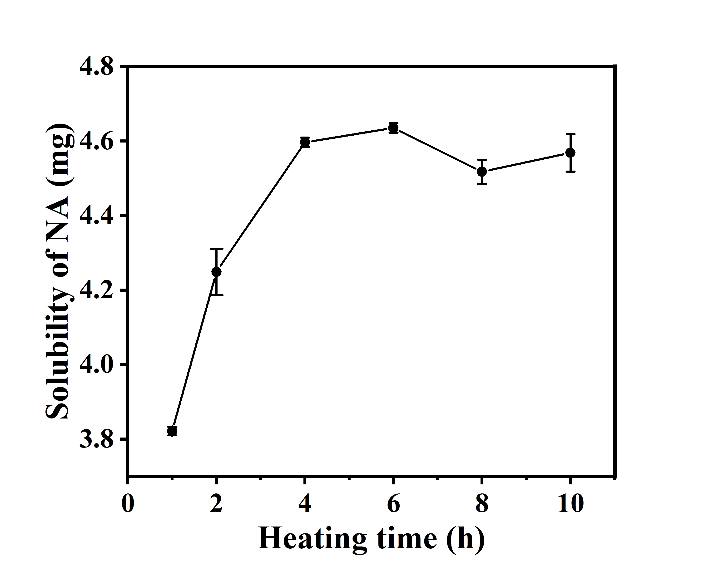


**Figure S3.** The effect of heating duration to solubility of NA.


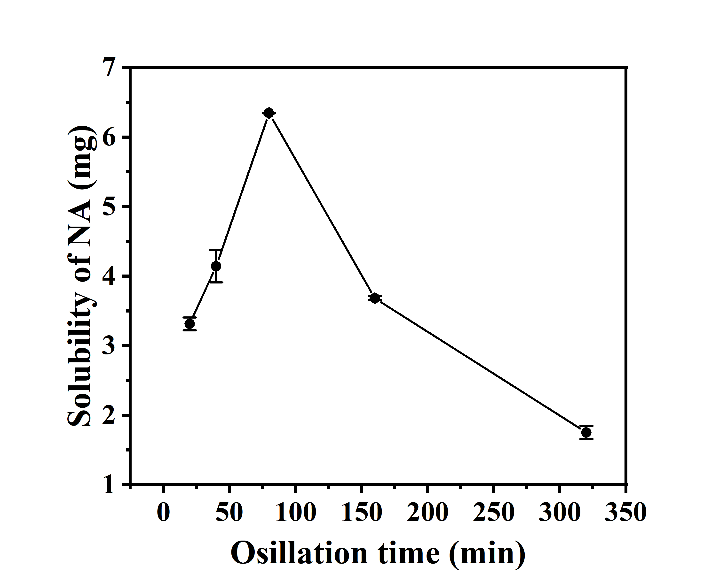


**Figure S4.** The effect of oscillation time to solubility of NA.


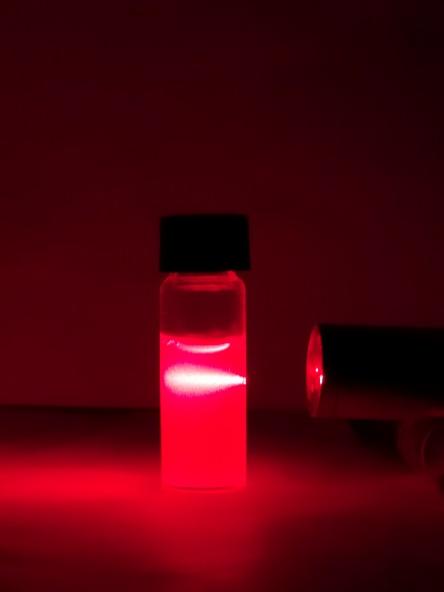


**Figure S5.** Tyndall phenomenon of NA suspension.


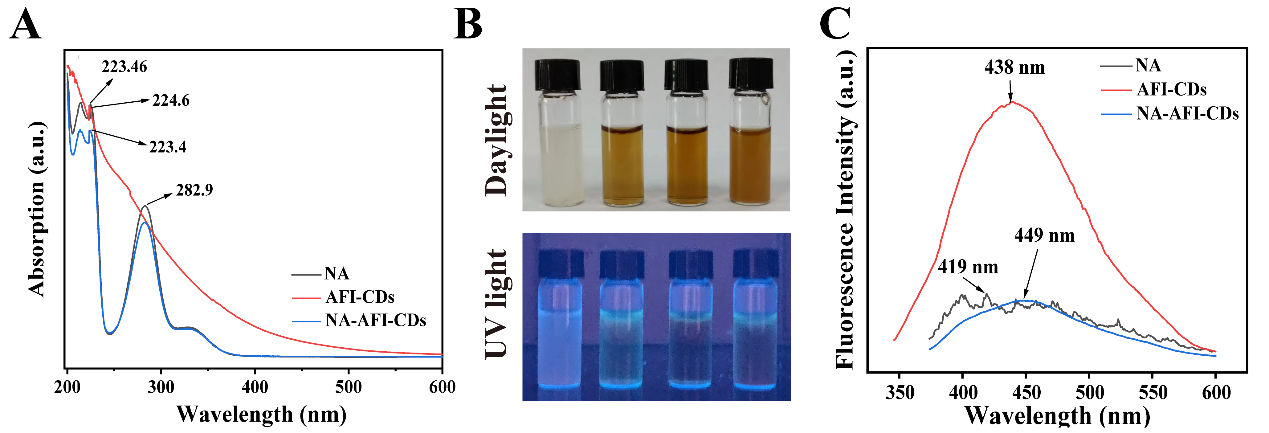


**Figure S6.** (A) The UV-vis spectra of NA, AFI-CDs and NA-AFI-CDs. (B) Photographic images of CDs fractions of NA, AFI-CDs, NA-AFI-CDs, and physical mixture (in order from left to right) under daylight and 365 nm UV illumination. (C) Fluorescence spectra of NA, AFI-CDs and NA-AFI-CDs at the same excitation wavelength.


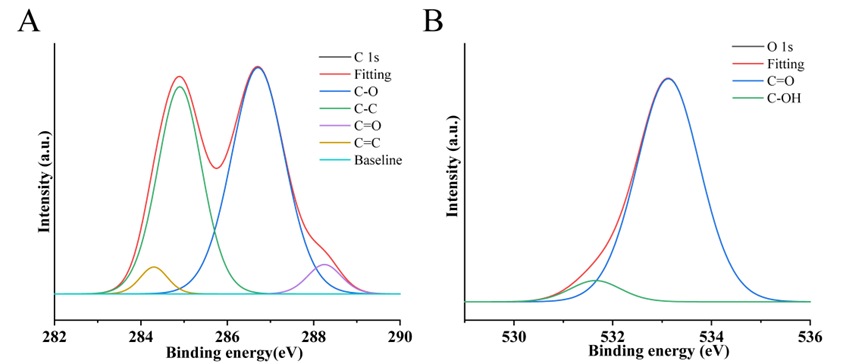


**Figure S7.** The XPS spectra of NA. (A) The C 1s high-resolution and (B) O 1s high-resolution spectra.


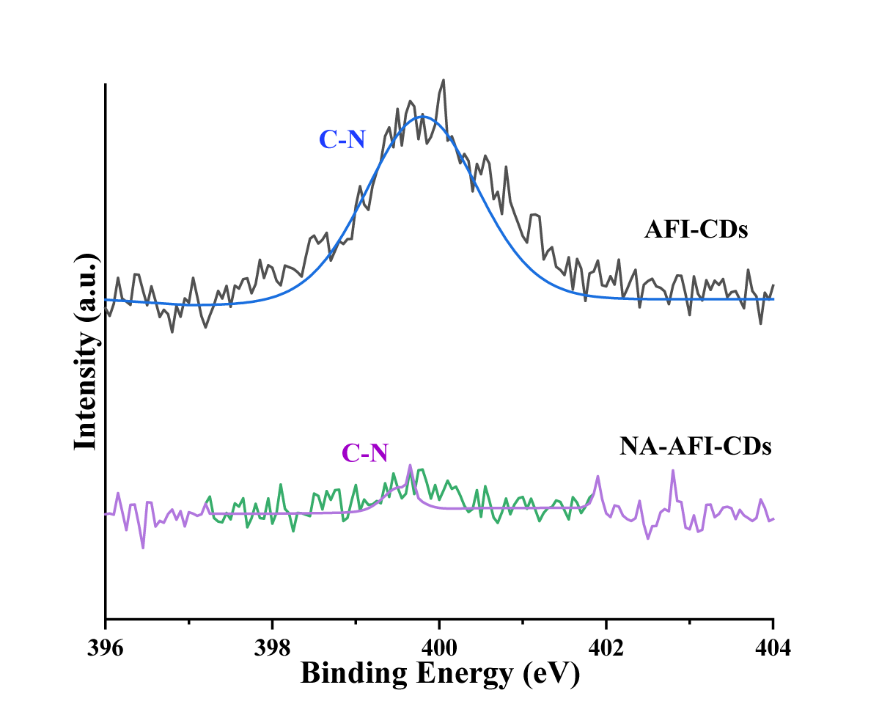


**Figure S8.** The N 1s XPS spectra of AFI-CDs and NA-AFI-CDs.


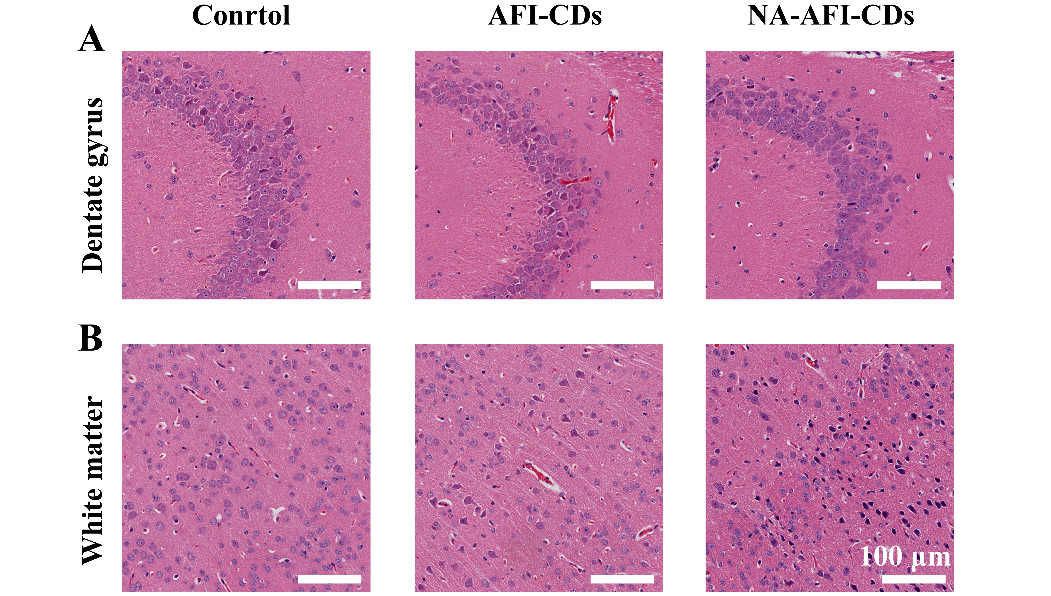


**Figure S9**. Brain histopathological sections of control, AFI-CDs and NA-AFI-CDs groups. (A) Dentate gyrus. (B) White matter.


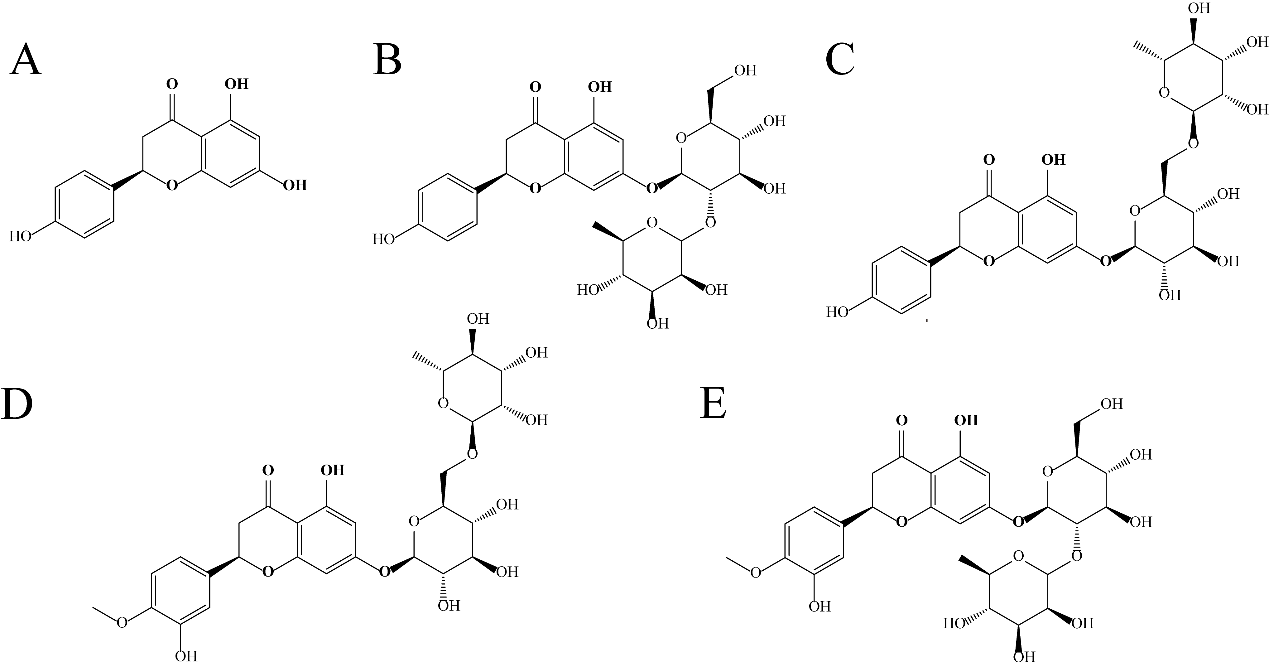


**Figure S10.** The structure of the (A) naringenin, (B) naringin, (C) narirutin, (D) hesperidin and (E) neohesperidin.


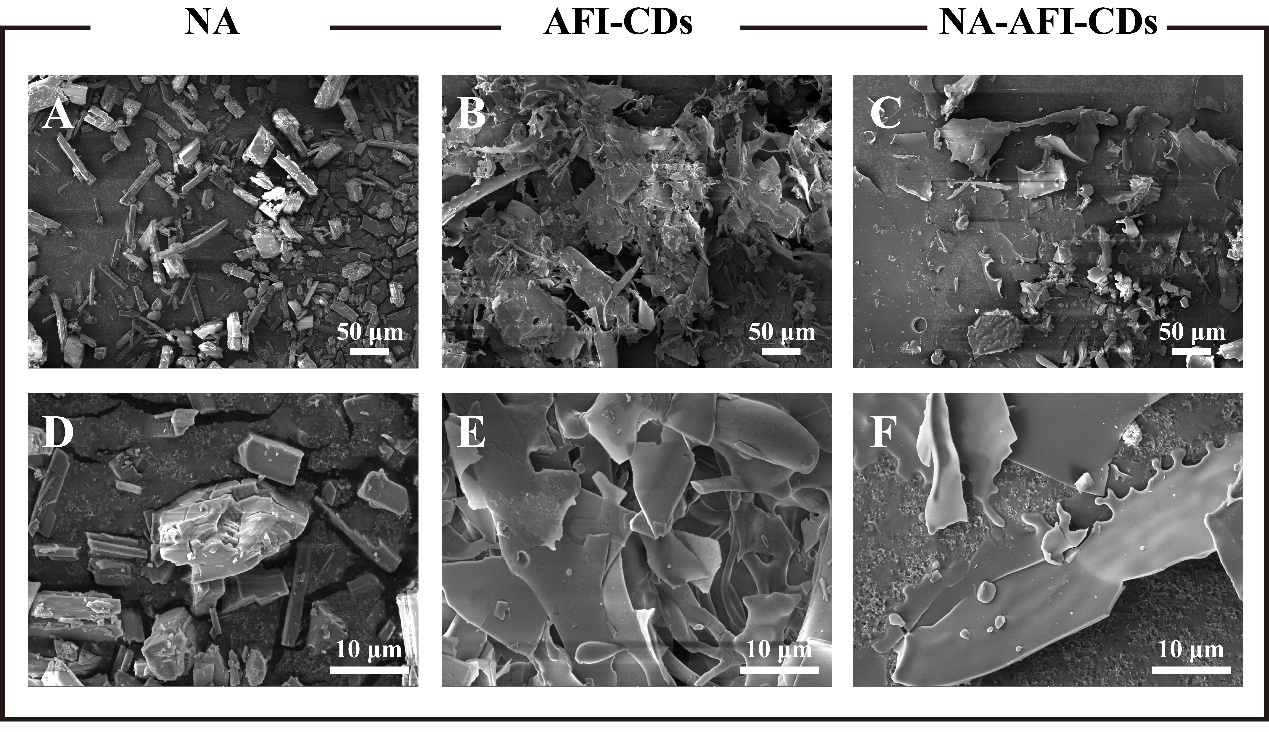


**Figure S11**. FE-SEM images of NA (A, D), AFI-CDs (B, E), and NA-AFI-CDs (C, F) in different scales (50 μm for A, B, and C; 10 μm for D, E, and F).

# Supplementary Tables

| **No.** | **Name of precursors** | **The integral area of NA** | **SE** |
| --- | --- | --- | --- |
| 1 | *Aurantii fructus immaturus* | 44775.7 | 216.7 |
| 2 | *Granati pericarpium* | 40302.5 | 195.1 |
| 3 | Monomer (flavonoids) | 27977.9 | 135.4 |
| 4 | *Trachycarpi petiolus* | 25011.9 | 121.1 |
| 5 | *Puerariae lobatae radix* | 24653.8 | 119.3 |
| 6 | *Scutellariae radix* | 24112.6 | 116.7 |
| 7 | *Vaccariae semen* | 23888 | 115.6 |
| 8 | *Astragali radix* | 23575 | 114.1 |
| 9 | *Glycyrrhizae radix et rhizoma* | 23560.7 | 114.0 |
| 10 | *Junci medulla* | 9554.4 | 46.2 |
| 11 | *Acrantii fructus* | 6698.7 | 32.4 |
| 12 | *Eucommiae cortex* | 6620.9 | 32.0 |
| 13 | *Poria* | 5050 | 24.4 |
| 14 | Monomer (flavonoids) | 4446 | 21.5 |
| 15 | *Citri reticulatae pericarpium* | 3668 | 17.7 |
| 16 | *Rehmanniae radix* | 2599.2 | 12.6 |
| 17 | *Gardeniae fructus* | 2597.2 | 12.6 |
| 18 | *Crataegi fructus* | 2191.5 | 10.6 |
| 19 | Monomer (polyphenols) | / | / |
| 20 | *Ailanthi cortex* | / | / |
| 21 | *Coptidis rhizoma* | / | / |
| 22 | *Sophorae flos* | / | / |
| 23 | *Sanguisorbae radix* | / | / |
| 24 | *Rhei radix ehizoma* | / | / |

**Table S1.** The previous experiment to explore the optimal solubilization effect on NA by different herb-derived CDs.

| AFI-CDs concentration (μg/mL) | Total Solubility of NA (mg) | DLE (%) | SE (fold) |
| --- | --- | --- | --- |
| 7.8125 | 0.02 | 0.20 | 1.68 |
| 15.125 | 0.89 | 11.12 | 31.36 |
| 31.25 | 2.10 | 26.25 | 72.50 |
| 62.5 | 3.96 | 49.46 | 135.57 |
| 125 | 4.25 | 53.08 | 145.42 |
| 250 | 4.74 | 59.24 | 162.16 |
| 500 | 6.34 | 79.31 | 216.72 |
| 750 | 6.17 | 77.08 | 210.65 |
| 1000 | 5.96 | 74.52 | 203.70 |

**Table S2.** The effect of different concentrations of AFI-CDs.

| NA dosage (mg) | Total Solubility of NA (mg) | DLE (%) | SE (fold) |
| --- | --- | --- | --- |
| 2 | 0.75 | 43.47 | 26.54 |
| 4 | 2.53 | 62.74 | 87.04 |
| 6 | 3.99 | 69.98 | 136.68 |
| 8 | 6.17 | 77.08 | 210.66 |
| 10 | 5.95 | 61.06 | 203.22 |
| 12 | 4.53 | 37.56 | 154.93 |

**Table S3.** The effect of different NA dosage.

| Temperature (℃) | Total Solubility of NA (mg) | DLE (%) | SE (fold) |
| --- | --- | --- | --- |
| 40 | 2.42 | 30.28 | 83.44 |
| 60 | 4.70 | 53.47 | 160.83 |
| 80 | 6.34 | 78.92 | 216.72 |
| 100 | 4.93 | 58.92 | 168.51 |
| 120 | 4.86 | 55.68 | 166.11 |
| 140 | 4.58 | 55.91 | 156.72 |

**Table S4.** The effect of different heating temperatures.

| Temperature time (h) | Total Solubility of NA (mg) | DLE (%) | SE (fold) |
| --- | --- | --- | --- |
| 1 | 3.82 | 47.77 | 130.97 |
| 2 | 4.25 | 53.04 | 145.49 |
| 4 | 4.60 | 57.03 | 157.31 |
| 6 | 4.63 | 57.94 | 158.62 |
| 8 | 4.52 | 56.46 | 154.61 |
| 10 | 4.57 | 57.10 | 156.35 |

**Table S5.** The effect of different heating duration.

| Skating time (min) | Total Solubility of NA (mg) | DLE (%) | SE (fold) |
| --- | --- | --- | --- |
| 20 | 3.31 | 41.43 | 113.75 |
| 40 | 4.14 | 51.78 | 141.88 |
| 80 | 6.34 | 79.31 | 216.72 |
| 160 | 1.75 | 46.04 | 60.63 |
| 320 | 3.68 | 21.89 | 126.30 |

**Table S6.** The effect of different oscillation time.

| Group name | C (%) | O (%) | N (%) |
| --- | --- | --- | --- |
| NA | 67.41 | 32.01 | 0.59 |
| AFI-CDs | 70.2 | 27.27 | 2.37 |
| NA-AFI-CDs | 67.33 | 32.47 | 0.2 |
| N+A mix | 66.01 | 32.99 | 0.9 |

**Table S7.** The elemental composition of NA, AFI-CDs, NA-AFI-CDs and N+A mix (physical mixture).
